# Marital Relationships and Mental Health Among Healthcare Workers

**DOI:** 10.1192/j.eurpsy.2026.11817

**Published:** 2026-07-31

**Authors:** I. Sellami, A. Abbes, A. Feki, M. L. Masmoudi, M. Hajjaji, K. Jmal Hammami

**Affiliations:** 1 occupational medicine; 2 Rheumatoloy, Hedi Chaker Hospital, sfax, Tunisia

## Abstract

**Introduction:**

Marital relationships play a critical role in the psychological well-being of individuals, including healthcare workers exposed to occupational stress. The quality of marital relationships can significantly influence personal mental health and overall well-being.

**Objectives:**

Our study aimed to explore the effect of marital relationships on mental health among healthcare workers.

**Methods:**

We conducted a descriptive, analytical, cross-sectional survey among workers. We collected sociodemographic, professional characteristics, and perceived stress using the perceived stress scale. Satisfaction of marital relationships were rated on a 0–10 scale, with 0 representing very poor and 10 representing excellent relationship.

**Results:**

Our population comprised 57 HCWs, 52.6% of whom were female. The mean age of participants was 36.1 ±10.2 years. The mean of the tenure of job was 15 ±11.5 years. The mean of job satisfaction was 3.4 ±1.2. The average marital relationship satisfaction score was 8.2±1.9. The mean perceived stress was 19.2 ±11.5. We found 3.5% of participants presented low stress, 93% moderate stress and 3.5% presented high perceived stress. High perceived stress was significantly associated with lower satisfaction in marital relationships (r = -0.5, p = 0.005).

**Conclusions:**

Perceived stress among healthcare workers is inversely associated with marital relationship satisfaction, underscoring the importance of addressing personal and relational well-being to support mental health in HCWs.

**Disclosure of Interest:**

None Declared

